# Efficacy and Safety of Vagus Nerve Stimulation in Lennox–Gastaut Syndrome: A Scoping Review

**DOI:** 10.3390/children11080905

**Published:** 2024-07-27

**Authors:** Debopam Samanta

**Affiliations:** Division of Child Neurology, Department of Pediatrics, University of Arkansas for Medical Sciences, Little Rock, AR 72202, USA; dsamanta@uams.edu; Tel.: +1-(501)364-1850; Fax.: +1-(501)364-6077

**Keywords:** Lennox–Gastaut syndrome (LGS), vagus nerve stimulation (VNS), drug-resistant seizures, neuromodulation therapy, seizure outcomes, quality of life

## Abstract

Lennox–Gastaut syndrome (LGS) is a severe developmental and epileptic encephalopathy characterized by drug-resistant seizures, cognitive impairments, and abnormal electroencephalographic patterns. Vagus nerve stimulation (VNS) is a widely used neuromodulation therapy for LGS, but its effects on seizure outcomes, different seizure types, non-seizure outcomes, and adverse events in this population have not been comprehensively reviewed. To conduct a scoping review on the use of VNS in LGS, a literature search was performed in PubMed, OVID, Web of Science, and Embase from inception to 9 June 2024, using relevant keywords and without restrictions on study design. The search yielded forty eligible studies (twenty-four retrospective cohorts, fourteen prospective cohorts, and two registry analyses) comprising 1400 LGS patients treated with VNS. No randomized controlled trials were identified. Across studies, the median seizure reduction ranged from 20.6% to 65%, with 0% to 100% of patients achieving a ≥50% seizure reduction. No consistent preoperative biomarker of VNS responsiveness was identified in LGS. Although inconsistent among different studies, tonic, atonic, and tonic–clonic seizures responded best, while focal seizures responded worst. Improvements in seizure severity, alertness, and quality of life were reported in some studies, but cognitive and adaptive functioning generally remained unchanged. Adverse events were mostly mild and transient, including hoarseness, cough, and paresthesia. Device-related complications and infections were uncommon. In conclusion, further research is needed to better understand VNS’s position in the evolving LGS treatment landscape and its cost effectiveness.

## 1. Introduction

Lennox–Gastaut syndrome (LGS) is a prevalent developmental and epileptic encephalopathy (DEE), with around 20% of patients with infantile epilepsy progressing to LGS [1,2]. This syndrome is characterized by [1] the presence of various drug-resistant seizures beginning before 18 years of age [including tonic seizures and potentially atypical absence, atonic, myoclonic, focal impaired awareness (FIAS), generalized tonic–clonic seizures (GTCS), and epileptic spasms]; Ref. [2] cognitive and frequently behavioral impairments; and [3] diffuse slow spike-and-wave and generalized paroxysmal fast activity on electroencephalography (EEG) [3]. LGS is associated with a high rate of seizure emergencies, including nonconvulsive status and high premature mortality [2,4]. The high frequency of seizures, intellectual disabilities, and behavioral impairments result in a low quality of life and significant daily functional support needs for individuals with LGS, imposing a heavy burden on caregivers, which manifests as fatigue, sleep disturbances, social isolation, poor mental health, and financial challenges [5]. Despite polytherapy, pharmacoresistance is common in LGS, leading to frequent use of non-pharmacological treatments such as epilepsy surgery, dietary therapy, and neuromodulation [4].

Vagus Nerve Stimulation (VNS) Therapy^®^ was the first FDA-approved neuromodulation therapy for drug-resistant epilepsy (DRE). The European Community approved VNS in 1994, followed by the FDA in the USA in 1997 for patients over 12 years old with pharmacoresisant focal seizures [6]. A later evidence-based guideline from the American Academy of Neurology (AAN) recommended its use in children with either focal or generalized epilepsy, based on 14 Class III studies, pooling data from 481 children with a 55% responder rate [7]. This guideline also provided a level C recommendation for VNS in patients with LGS based on four Class III studies [8,9,10,11]. A pooled analysis of 113 patients with LGS showed a 55% (95% CI 46–64%) responder rate [7].

Currently, VNS is among the most frequently performed pediatric neurosurgical procedures, with LGS being the leading cause of pediatric epilepsy treated with VNS [12]. Consequently, some systematic reviews and meta-analyses have calculated the seizure responsiveness rate of LGS following VNS, with or without comparisons to other surgical treatments like corpus callosotomy [13,14,15]. However, there has been a lack of exploration into other important outcomes, specifically VNS’s effect on various seizure types, non-seizure outcomes including quality of life, and adverse effects [1]. A holistic exploration of these outcomes is crucial for clinical decision-making, as VNS and other nonpharmacological options do not render patients seizure-free, with approximately half of the patients being responders [16]. Therefore, physicians and caregivers need to consider these additional outcomes. Additionally, emerging neuromodulation techniques with diverse attributes, such as deep brain stimulation (DBS) and responsive neurostimulation (RNS), are increasingly accepted for treating LGS [17]. This evolution necessitates a thorough review of all attributes of VNS to determine the best neuromodulation treatment approach that aligns with the preferences of patients, families, and physicians.

## 2. Materials and Methods

To allow a comprehensive analysis of the efficacy and safety of VNS in LGS, we chose to undertake a scoping review approach rather than a systematic review. Our goal was to broadly examine the literature and address several previously unexplored research questions following a PICO framework (Table 1).

**Figure 1:**
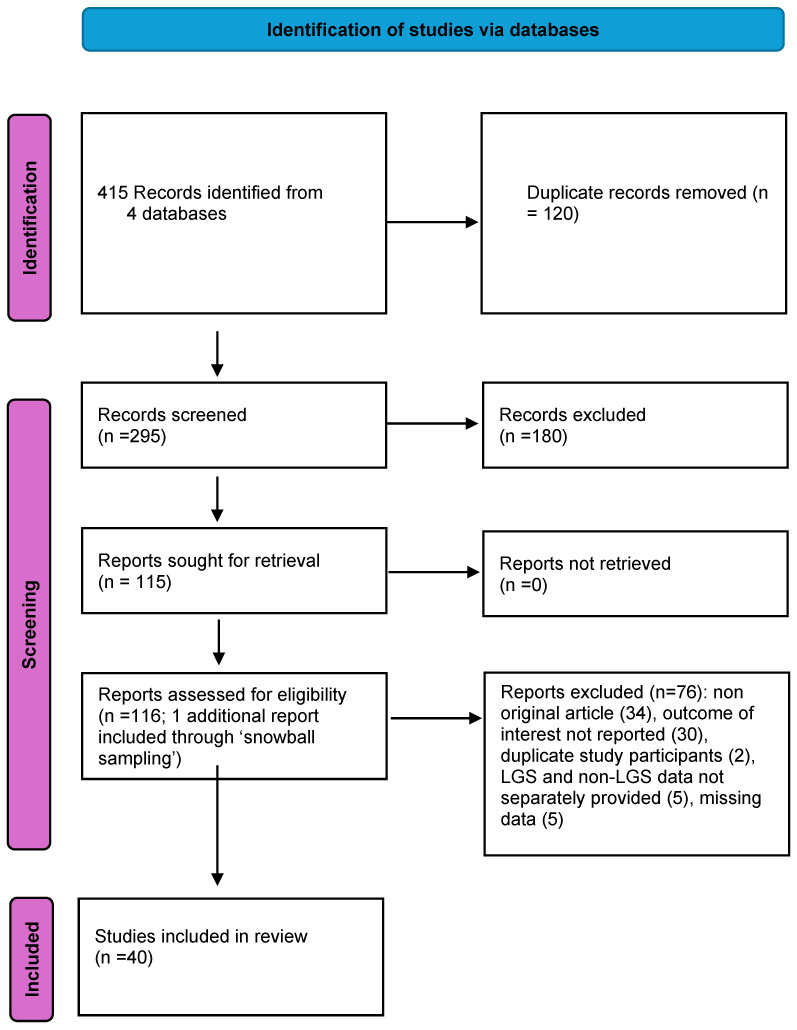
PRISMA (Preferred Reporting Items for Systematic Reviews and Meta-Analyses) flowchart.

The review followed the steps described by the PRISMA guidelines [18], Supplementary Materials. (Figure 1) For this scoping review, we utilized multiple searchable databases, including PubMed, OVID, Web of Science, and Embase from inception to 9 June 2024, using relevant keywords and without restrictions on study design. Our search strategy was structured as follows: ((terms for Lennox–Gastaut syndrome OR Lennox–Gastaut OR LGS) AND (VNS OR vagal nerve stimulation OR vagus nerve stimulation)). We also checked the reference lists of eligible studies and relevant systematic reviews using a ‘snowball sampling’ approach. The databases were searched from their inception to 9 June 2024. We imposed no restrictions on study design.

Abstracts identified through the searches were screened using predefined inclusion and exclusion criteria (Table 1) tailored to address the research questions. Relevant studies were retrieved in full-text format and further screened for the outcomes of interest. The criteria for inclusion and exclusion remained the same as those used for abstract screening, with the additional exclusion criteria detailed in Figure 1. Studies were excluded if they included patients with LGS but did not provide a specific number for the LGS group or did not report specific outcomes for the LGS subgroups (at least one outcome related to seizures, non-seizure events, or adverse events had to be reported separately for LGS). Single case reports of adverse effects were not included in the assessment but were mentioned in the text. Given that the studies were expected to be small, heterogeneous, and largely non-comparative, no formal statistical synthesis or quality assessment was planned. All qualitative and quantitative data were summarized in a narrative synthesis.

## 3. Results

### 3.1. Study Characteristics

The search yielded forty eligible studies (twenty-four retrospective cohorts, fourteen prospective cohorts, and two registry analyses) comprising 1400 LGS patients treated with VNS (Table 2). Multiple studies were reported from USA, Canada, Sweden, Italy, Brazil, Korea, and Norway. No randomized placebo-controlled trial (high vs. low stimulation) has specifically evaluated the efficacy of VNS in LGS. The largest study included a registry study with 564 LGS patients who were compared to 1128 non-LGS patients [19]. Another large registry study included 552 patients (167 with outcomes data) with LGS from the VNS Patient Registry in the United States in 2001 [20]. The largest single-center study included 62 patients [21]. The largest multicenter study, a retrospective open-label study conducted at 11 centers across the European Union, consisted of 146 LGS patients [22]. Many studies in this review included different epilepsy groups, with LGS often representing the largest subgroup [23]. Some studies compared VNS efficacy with corpus callosotomy [9,24]. Others compared LGS with other epilepsy groups, such as genetic generalized epilepsy, other DEEs, or severe epilepsy with multiple independent spike foci [19,25,26]. One study reported seizure outcomes in LGS for a range of therapeutic modalities, including VNS [27].

### 3.2. Seizure Outcomes

#### 3.2.1. Seizure Reduction

The seizure outcomes were reported as percentages of mean or median seizure reduction. Some studies only reported seizure responders, defined as patients with more than 50% seizure reduction [23]. Few studies utilized the McHugh Classification, which is modeled on the Engel classification but specifically tailored for use in VNS, including subdivisions to capture changes in ictal or postictal severity [28]. 

The efficacy of VNS varied across studies, likely due to differences in patient populations, study designs, and follow-up periods. Seizure responder rates (≥50% seizure reduction) varied widely, ranging from 0% to 100% [9,10,11,21,29,30,31,32,33,34,35,36,37,38]. The overall median seizure reduction for LGS patients ranged from 20.6% to 65% across different studies [21,39]. One study reported 11% of LGS patients achieving ≥ 75% seizure reduction (Class II responders) at 5 years [10]. Another study reported that five out of six patients with >90% seizure reduction [40]. Seizure-free outcomes were noted in 5.2% (range 2.3–9.2%) of cases [14]. 

One study reported differences in seizure responsiveness between day and night, with a greater effect during the daytime and complete abolition of daytime drop attacks [32].

The effectiveness of VNS in LGS patients was noted to be comparable to other DRE patients [41]. One study showed a seizure responder rate of 57.5% in LGS compared to 61.5% in other groups after 24 months of VNS implantation [19]. The median seizure reduction was 64.3% in the LGS compared to 66.7% in the non-LGS group [19]. Another study showed median seizure reduction of 52.1% in LGS compared to 57.1% in the non-LGS group with no statistical difference between these groups [21]. LGS was also compared with genetic generalized epilepsy (GGE). Suller Marti et al. showed that 41.7% of patients in the LGS group had an overall seizure reduction of 50% or more, compared to 64.7% in the GGE group, without statistical significance between the groups [26]. One study suggested that VNS may have superior efficacy in treating focal epilepsy compared to LGS [42]. Another study indicated a better seizure freedom rate and a greater than 75% seizure reduction rate in GGE compared to LGS following VNS [26].

#### 3.2.2. Seizure Types and Responsiveness to VNS 

The efficacy of VNS in reducing specific seizure types in LGS patients varies widely and inconsistently across different studies and among patients within a single study [43]. Specific seizure types in LGS that showed significant reductions included atonic seizures (up to 64.6% median reduction) and tonic seizures (50%) [10,24,33]. Dibue reported a 90% decrease in drop seizures (atonic and other seizure types leading to falls) over 24 months, with 78% of patients experiencing a 50% or greater reduction in drop seizures [19]. In general, GTCS showed a less significant reduction than atonic or tonic seizures [14]. One study reported only a 33.3% reduction in tonic–clonic seizures (TCS) [21]. However, contrasting findings were noted in other studies [8,24]. Cukiert reported no change in tonic and atonic seizures following VNS, but found significant improvement in GTC seizures [24]. Improvement in TCS was also noted in other studies, with 74.1% and 60% of patients experiencing a 50% or greater reduction in focal to bilateral TCS and bilateral TCS, respectively. Other studies showed the most significant reductions in GTCS [33,44]. However, efficacy against TCS might diminish during follow-up [19]. Compared to tonic, atonic, and TCS, FIAS had a less robust reduction of 33% [21]. Frost et al. showed FIAS decreased by only 20% after 1 month and 23% after 3 months [33]. Some studies did not report absence seizures due to difficulty in counting [21]. However, other studies reported excellent efficacy against absence seizures [24,33,44]. Effects on spasms and myoclonic seizures were infrequently reported. One study showed spasms reduced by a median of 21.7% [21]. Seven of twelve LGS patients reported clinically significant reductions in myoclonic jerks, and one reported becoming free of this seizure type [21]. Significant reduction in myoclonic seizures was also reported in other studies [24,37]. One study reported the effects of VNS on residual seizures of various types after corpus callosotomy [45]. The best response was noted in tonic seizures, while no response was seen in atonic seizures [45]. Residual myoclonic seizures after callosotomy responded better than atypical absence seizures [45]. 

#### 3.2.3. Variables Affecting Responsiveness

Some studies examined the different variables associated with LGS and its responsiveness to VNS. In the LGS group, Cersosimo did not find any differences between symptomatic and cryptogenic patients regarding the severity of intellectual disability or seizure reduction [11,46]. Additionally, there were no differences in outcomes between patients who had previously had West syndrome and those who had not [46]. History of prior callosotomy did not alter the responsiveness [20]. Age younger than 12 vs. older did not show any improved responsiveness [21,33]. A change in heart rate variability (HRV) reported in a patient with LGS who initially had minimal HRV but showed improved variability following VNS [47]. HRV was also proposed as a measure to detect VNS responsiveness. However, one study showed no difference in pre-ictal HRV parameters between VNS responders and non-responders, despite a significant increase in high-frequency (HF) power, which indicates parasympathetic tone, significantly increased in the pre-ictal period for both groups [48]. 

#### 3.2.4. Immediate and Long-Term Efficacy

Several studies have reported an immediate effect before stimulation is turned on and also longitudinal follow-up over several years. Cukiert et al. observed an implantation effect lasting an average of 20.2 days in 14 out of 24 children [24]. However, this has not been consistently reported, and most studies indicated greater efficacy between 3 and 12 months of stimulation, with progressive improvement over the first 24 months also reported.

In a retrospective multicenter study of 50 LGS patients treated with VNS, the median seizure reduction was 42% after one month, increasing to 58.2% after three months [33]. Progressive improvement over 24 months was reported in a study that showed a mean reduction in seizure frequency of 8% at 3 months, 33% at 12 months, and 50% at 2 years [42]. Another study reported seizure responder rates of 28.5%, 32.9%, and 39.1% at 6, 12, and 24 months, respectively [22]. A separate study on closed-loop VNS showed seizure responder rates of 55%, 67.7%, and 65% at 6, 12, and 24 months, respectively [49]. Longer follow-up durations of up to 5 years have also been reported. In one study, seizure reduction at 6, 12, and 60 months was 17.7%, 35.5%, and 30.6%, respectively [21]. This study noted a statistically significant increase in seizure reduction from 6 to 24 months (*p* = 0.006) and persistence of efficacy but no further improvement since 24 months of follow-up. Additionally, a progressive increase in seizure responsiveness greater than 75% was reported, from 15% at 1 month to 35% at 6 months, and then 38% at 12 months [33]. However, one study reported a reversal of response in bilateral TCS during long-term follow-up [19].

#### 3.2.5. Impact on Seizure Severity and Status Epilepticus

Approximately half of the patients with LGS experience prolonged seizures, seizure clusters, and status epilepticus [50]. VNS studies have reported reductions in seizure duration, ictal and postictal severity, and seizure clustering in a significant proportion of LGS patients [10,21,22,33]. One study noted a decrease in seizure severity scores measured with the adapted Chalfont severity scales [8]. Another study observed a significant decrease in hospitalizations and episodes of status epilepticus in some patients after VNS implantation [32]. Another study showed seizure-related hospitalization decreased from 96.6% to 51.7% after VNS implantation [26]. Most responders in the study experienced milder, shorter seizures with faster recovery times [32]. Nagarajan et al. reported that four out of five patients had over 50% seizure reduction, with most experiencing reduced duration and severity of seizures, and two had reduced diazepam use [51]. Lundgren et al. found that two out of four patients had decreased seizure severity scores [52]. Frost et al. showed that around 30% of patients had reduced postictal severity and seizure clustering [33]. Mikati et al. reported that three out of six patients had improvement in seizure severity, and seizure duration decreased in two patients [53]. Seizure severity reduction may be more pronounced in LGS than in other groups. One study reported that DEE patients (62 out of 105 had LGS) showed greater improvement in ictal or postictal severity (43.8% vs. 28.3%, *p*  =  0.006) compared to patients without intellectual disability, possibly leading to a higher rate of continuing use of VNS at 5 years [21].

#### 3.2.6. Standard vs. Rapid Cycling, Magnet Stimulation, and Autostimulation

The optimal device parameters remain uncertain and do not show clear associations with treatment outcomes [33]. Rapid cycling has not been shown to have additional benefit than standard stimulation [54]. Although additional magnet-activated stimulation for seizures associated with LGS has not consistently shown efficacy [34,51], one study demonstrated a reduction in seizure duration or severity in 61.0% of 105 patients with DEE, including 62 with LGS [21].

A newer method employing closed-loop autostimulation, which delivers electrical stimulation promptly in response to ictal tachycardia, has been found effective without increasing the average charge per day [55]. Winston compared closed-loop versus open-loop VNS devices and observed greater seizure reduction and more favorable clinical outcomes with closed-loop devices at 9 months, though not at 24 months, across the entire cohort [56]. However, there were no significant differences noted in the LGS subgroup during either follow-up period. Conversely, Abdelmoity et al. reported that auto-stimulation may offer additional benefits over standard stimulation. In a study involving 71 children with LGS, 55% achieved >50% seizure reduction at six months, 67.7% at 12 months, and 65% at 24 months with autostimulation VNS [49]. At 12 months, 11% were completely seizure-free, and at 24 months, 17% remained seizure-free [49]. Moreover, when transitioning from traditional to auto-stimulation models, 60–70% experienced further reductions in seizure frequency from baseline [49].

#### 3.2.7. Cognitive and Adaptive Functioning, Quality of Life Outcomes

Almost all individuals with LGS have cognitive and behavioral impairments of varying severity, which limits standardized assessment. The severity of impairment may also preclude formal testing. However, some VNS studies have attempted to use various cognitive and psychological/behavioral instruments. Hallbrook et al. assessed cognitive development using the Bayley Scales of Infant Development and the Wechsler Preschool and Primary Scale of Intelligence (WPPSI), while Parker et al. utilized the Vineland Adaptive Behavior Scale and the Wellcome Quality of Life (QoL) Assessment, specifically designed for LGS [29,57]. Parker also employed the British Picture Vocabulary Scale (BPVS), the Leiter International Performance Scale, and Conner’s Parent/Teacher Rating Scales [57]. Aldenkamp used Dutch scales for provider assessments, including the Dutch version of the Bayley Developmental Scale, the Dutch version of the McCarthy Developmental Scale, and other scales to measure independence, communication skills, task acceptance, social sensitivity, and behavior disorders [39]. Mood was evaluated using a Dutch scale. Similar detailed evaluations were conducted by Majoie et al. [8].

Many LGS patients cannot complete age-appropriate test batteries. For example, one study reported that only one subject out of twenty-four could undergo formal psychological evaluation [24]. Even composite scaled scores from tests suitable for developmental age may be less useful due to floor effects (test scores falling ≥ 3 standard deviations below normalized sample means), necessitating the tracking of raw scores. Due to these difficulties, several studies relied on indirect indicators such as parental or provider reports using simple scales or questionnaires. For example, Frost utilized provider assessments employing a 5-point scale, while other studies used various tools like the QOLIE-31 questionnaire and the Child Epilepsy Questionnaire Parental Form (CEQ-P) [24,33,46,53]. Different studies used various scales, including the parental visual analog scale [29,52], a parental 3-point scale [51], a Likert scale [25], and a parental global evaluation score [40].

Most studies did not observe significant changes or deterioration in cognitive measures or adaptive functioning [8,25,31,39]. However, one study reported a mild positive change in mental age during follow-up, with the treatment effect being most prominent in the group with the highest mental age at baseline [39]. Some studies reported improvements in quality of life, alertness, social communication, and reductions in behavioral disturbances in certain patients [24,31,46,52,58,59]. Frost et al. found improvements in alertness for 50–60% of patients, over 20% for verbal abilities, and less for memory/ambulation during 3–6 months of the follow-up period [33]. Cukiert et al. observed improvement in attention (based on 18 items Swanson, Nolan, and Pelham Questionnaire IV) in 21 out of 24 children and reported quality of life/health improvements in up to 50% (mean 25%) of 20 children [24]. Another study of closed-loop VNS showed at least a 50% improvement rate in one or more quality-of-life measures (alertness, sleep, development, academic performance, and attention) in most patients [49]. Four studies indicated that changes in quality of life, cognition, and behavior were observed independently of seizure reduction outcomes [24,25,33,39]. One study reported no significant changes in cognitive level and adaptive behavior scores at group level following 1 and 3 years of stimulation, but four patients had clinically relevant improvement in adaptive behavior (at least 5-point increase in standard scores) mainly due to increased alertness and a better social reciprocity [25]. One study reported that six out of seven patients showed improvement on the Clinical Global Impression (CGI) improvement scale, with two patients each experiencing marked, moderate, and minimal improvements [59]. Katagiri et al. reported that VNS responders had better conversational ability than non-responders [45]. However, consistent improvements in quality of life were not observed across all studies. For instance, Aldenkamp et al. did not find significant changes in quality-of-life measures [39]. One study reported mood decline in one out of twenty-four patients [33], and another study reported behavioral worsening in two out of ten patients [32].

One study demonstrated reduced direct healthcare (medication and hospitalization) and non-healthcare (traveling) costs following VNS [8]. The number of days of reduced functioning of the child was significantly less compared with the baseline period [8]. The assessed cost-effectiveness ratio was EUR 16.93 per reduction in one seizure [8].

### 3.3. Adverse Events

Adverse events were not specifically reported in all studies [39]. However, most studies noted no major complications [9,24,25,29,31,32,45,46,51]. One study did report four serious adverse events, including three cases of status epilepticus and one sudden death [44]. Nevertheless, all-cause mortality and SUDEP rates for VNS therapy in LGS patients have not yet been reported. Some other studies mentioned side effects but did not distinguish between LGS and other epilepsy patients [21]. 

While the majority of patients experience some side effects [33], these are mostly mild and transient, such as hoarseness, cough, paresthesia (neck tingling), voice alterations, shortness of breath, pain, drooling, dyspepsia, decreased appetite, hiccups, and headache. Although some studies reported only 5–30% of these side effects [9,24,60], others reported 50–67% [10,32]. These side effects are not excessively high in LGS compared to other populations. One study showed the frequency of side effects similar in LGS vs. non-LGS groups (62.1% vs 64.7%) [26]. Side effects during VNS use are typically related to the “on” phase of stimulation and tend to diminish over time, usually disappearing within five years of implantation. Other unusual side effects include torticollis, inappropriate laughter, involuntary movement, urinary retention, aspiration, and lower facial paresis. A 16-year-old patient with LGS was reported to have symptomatic complete atrioventricular block due to VNS [61]. 

Children with cognitive impairments often pick at or touch wounds, increasing the risk of infection. However, less than 5% of LGS patients reported infections that required antibiotic treatment, and even fewer needed the external hardware removed and reinserted because of this [33]. One large study showed that two out of sixty-four patients developed an infectious complication [46]; in one case, the device was moved to the right side. To help these children, doctors have implanted the device in alternate locations, including under the pectoral muscles.

Device-related complications are less frequently reported in LGS literature. Orosz et al. tracked adverse effects from a patient complaint database and active post marketing surveillance, noting damage to the device’s lead (nineteen cases, 5.4%), medical device changes (fifteen cases, 4.2%), device removal (thirteen cases, 3.7%), device-related infections (nine cases, 2.5%), device-related complications (six cases, 1.7%), and issues related to the device lead (four cases, 1.1%) [22].

LGS patients are particularly susceptible to sleep-disordered breathing. A study of thirteen patients with DEEs, including six with LGS, showed a median arousal index of 29 per hour, with moderate to severe obstruction in 53.8% of patients and central apnea in 23% [62]. This is concerning because patients with VNS have been shown to have an increased incidence of obstructive and central apneas [63]. Although rare, some LGS patients have reported breathing difficulties following VNS, but a systematic exploration of sleep-disordered breathing has not been conducted. No studies reported the use of the SenTiva™ model, the newest VNS device with programmable stimulation that can be adjusted at different times of the day (e.g., day and night settings), to see if decreased stimulation parameters at night can alleviate sleep-disordered breathing.

Some patients experienced the emergence of a new seizure type following VNS that was not reported at baseline [19]. Status epilepticus and seizure worsening were reported in relation to higher settings. In four adult patients with LGS, increasing the output current to 2.00–2.25 mA and switching from standard stimulation (30 s on, 300 s off) to rapid stimulation (7 s on, 30 s off) caused worsened seizure frequency due to the increased total charge delivered per day [64]. This issue was resolved when the total charge delivered per day was reduced [64]. Additionally, one LGS patient experienced forced normalization (subacute psychosis) after turning off VNS, which had not provided significant benefit since its implantation [65]. However, the patient became seizure-free after VNS was turned off, suggesting a possible seizure-worsening effect of VNS that resolved when the device was deactivated, leading to psychosis [65].

## 4. Discussion

This scoping review, comprising 1400 patients with LGS from 40 studies, showed a wide range of seizure reduction and responder rates. Super responder and seizure freedom rates were less than 10%. Progressive improvement may be seen up to 24 months after implantation. The effectiveness of VNS in LGS patients was noted to be comparable to other DRE patients. Age, prior history of infantile spasms or corpus callosotomy, and specific etiology did not have any prognostic importance regarding responsiveness. Although rapid cycling may not have additional benefits over standard stimulation, magnet stimulation and closed-loop stimulation may provide additional efficacy. VNS may reduce seizure duration, ictal and postictal severity, seizure clustering, and seizure-related hospitalizations. The review assessed the adverse effects of VNS and noted primarily stimulation-related mild transient effects, with rare reports of infection and device-related complications.

In this scoping review, we also attempted to explore VNS’s effect on specific seizure types, as changes in total seizure counts might not reflect clinical improvement in LGS since different seizure types are not equally disabling. We noted that the efficacy of VNS in reducing specific seizure types in LGS patients varied widely and inconsistently across different studies. However, tonic, atonic, and tonic–clonic seizures responded best, while focal seizures responded worst [2,5,19]. Many studies did not specify particular seizure types for subgroups and likely only considered seizure reduction for the most disabling seizure types. Nevertheless, understanding the specific effects on seizure types may help with clinical decision-making in LGS patients, as many have distinct and most disabling seizure types. For example, atonic and TCS are usually more disabling than myoclonic and absence seizures, although the latter might be more frequent [21]. Although the most common seizure type in LGS is tonic seizures, TCS has specific significance regarding SUDEP, while atonic seizures are associated with severe craniofacial injuries due to falls. Although tonic, atonic, and tonic–clonic seizures were noted to respond best following VNS, the cause of the discrepancy between the effects of VNS on various seizure types in LGS is unclear. It may be related to study design, such as recall bias or errors in seizure classification or counting. In some studies, drop seizures were counted, which might capture either or both tonic and atonic seizures. Additionally, absence and myoclonic seizures are also difficult to count and thus prone to erroneous counting. Even some motor seizures, such as tonic seizures in LGS, are grossly underreported in the diary method compared to objective counting by VEEG [66].

The scoping review also explored VNS’s effect on cognitive and adaptive functioning, and quality of life outcomes in individuals with LGS. Although the use of age-appropriate psychological and cognitive test batteries was rare, indirect indicators such as parental or provider reports showed improvements in alertness and some measures of quality of life. However, there are no LGS-specific cognitive or QoL instruments, and the commonly used batteries varied widely. 

There are several limitations in this study. The first limitation is the heterogeneous study population and characteristics, with many outcomes preventing us from conducting a meta-analysis (meta-analysis of the seizure responder rate of VNS in this population has already been performed). The second limitation is that we did not find any randomized placebo-controlled trials (high vs. low stimulation) specifically evaluating the efficacy of VNS in LGS. All included studies were uncontrolled and open label, which may have led to an overestimation of the effectiveness of VNS. Although we did not perform a formal bias assessment, retrospective studies had issues with data quality, and prospective studies faced problems with attrition. The largest studies were based on observational registries with data collected prospectively but analyzed retrospectively. This design introduced limitations such as voluntary data collection by clinical staff without stringent monitoring, potentially affecting the consistency of seizure type classification and LGS diagnosis across different sites. Additionally, uncontrolled variability in anti-seizure medication (ASM) combinations and stimulation parameters may have introduced bias. The time frame for outcome assessment varies considerably among studies. In summary, most studies have small sample sizes and only meet the American Academy of Neurology Class III evidence criteria for therapeutic studies.

Despite these limitations, the study has several strengths. This report represents the most comprehensive review of VNS therapy in LGS patients so far and highlights potentially important differences across studies. Previous systematic reviews included 17 studies with 176–480 patients, whereas we included more than double the number of studies and approximately three times the number of individual patients [13,14,15]. Rather than comparing VNS with corpus callosotomy, this scoping review also delved deeper into non-seizure outcomes and adverse effects of VNS for a comprehensive outlook. As the emergence of RNS and DBS use may decrease the use of callosotomy, this review sets the stage for a comparison of VNS with other neuromodulatory approaches [17].

## 5. Conclusions

This scoping review assessed VNS use in LGS, highlighting significant knowledge gaps. Seizure outcomes following VNS in LGS vary widely, and identifying ideal candidates remains challenging despite exploring various biomarkers. As the epileptic connectivity in LGS becomes better understood, LGS-specific connectivity studies are necessary to preoperatively identify the best candidates for VNS [67]. The effectiveness of preoperative non-invasive VNS (e.g., transcutaneous auricular) in identifying candidates for invasive VNS has not been explored [68]. Although this review concentrated on invasive VNS, non-invasive VNS (nVNS) has also been examined in the context of epilepsy treatment [69]. nVNS presents several benefits, including simpler integration of control groups in clinical studies, reduced costs, fewer side effects, and potentially favorable effects on cognition [70]. Nevertheless, additional studies specifically targeting LGS are required to comprehensively assess its efficacy. 

Current treatment decisions for LGS are complex, involving ASM regimen modifications, callosotomy, or neuromodulation devices like DBS, RNS, or VNS, without specifically targeting seizure types [4]. Atonic seizures may respond better to callosotomy than VNS, but callosotomy has a higher complication rate and is less cost-effective [71]. The increasing availability of neuromodulation options complicates clinical decision-making, and the absence of comparative studies, especially for other seizure types, adds to this complexity. Conducting randomized head-to-head comparison studies is challenging, with only 21.3% of patients and caregivers willing to participate in randomization [72]. Due to the growing acceptance of DBS and RNS, deciding whether to use these before VNS is a consideration; however, a discrete-choice experiment indicated that VNS might be preferred over DBS by most clinicians [73]. For non-responders to VNS, DBS, or RNS may be effective alternatives, but further evaluation is needed to determine when these interventions should be used and which patients would benefit most from combined therapy [74,75]. As non-pharmacological approaches primarily provide palliation with rare seizure freedom, VNS studies should also focus on non-seizure outcomes, such as quality of life, cognitive function, psychiatric issues, and daily living, using LGS-specific standardized measures (which should be developed through a consensus approach). Understanding the cost-effectiveness of VNS compared to other treatments is crucial given limited healthcare resources [71]. More detailed economic evaluations can inform healthcare policy and reimbursement decisions.

## Figures and Tables

**Table 1:** PICO Framework for Evaluating VNS in LGS Patients.

PICO Element	Details
Patient Population	Patients diagnosed with Lennox–Gastaut syndrome (LGS), all ages. Exclusion: studies not specifically identifying LGS patients for any outcome measures.
Intervention	Vagus nerve stimulation (VNS) therapy
Control	Not required
Outcome Measures	- Seizure response: reduction in seizure frequency, responder rate, efficacy of VNS in reducing specific seizure types in LGS, variables affecting seizure responsiveness, short- vs. long-term efficacy
	- Other seizure outcomes: seizure severity, status epilepticus
	- Effects of different stimulation parameters
	- Non-seizure outcomes: cognitive function, adaptive behaviors, behavior, quality of life
	- Adverse events and complications
Inclusion Criteria	Studies reporting on VNS outcomes in LGS patients, regardless of publication date. English abstracts are required.
Exclusion Criteria	Studies where LGS patient numbers are not specifically identified. Non-English language studies without available translations.

**Table 2:** Characteristics and key findings of all 40 included studies.

Author	Country/Setting	Type of Study	N	Key Findings
Abdelmoity	Single center in the USA	Retrospective Cohort	71	A total of 55% of patients achieved greater than 50% seizure reduction at six months, 67.7% at 12 months, and 65% at 24 months. At 12 months, 11% of the patients were completely seizure free, and at 24 months, 17% were seizure free. Most families reported at least a 50% improvement in one or more quality-of-life measures. Common adverse events were dysphonia, paresthesia, and shortness of breath, which subsided by 24 months.
Alanazi	Single center in Saudi Arabia	Retrospective Cohort	4	Mean age of 13.8 ± 3.9 years; 2 (50%) patients achieved a reduction in seizure frequency of greater than 75% and 2 (50%) greater than 25%; ASM was unchanged in 3 and decreased in 1; swallowing difficulty ¼.
Aldenkamp	Single center in the Netherlands	Prospective Cohort	19	In 13 patients, LGS was confirmed; 5 had Lennox-like syndromes. Average age was 11.2 years. Average seizure reduction was 20.6% at 24 months. No significant deterioration in cognitive or quality of life measures. Mental age showed mild positive changes.
Benifla	Single center in Canada	Retrospective Cohort	10	A total of 4 out of 10 had a >50% seizure reduction. Subgroup analysis for non-seizure outcomes and complications were not provided.
Ben-Menachem	Single center in Sweden	Prospective Cohort	8	Average treatment time was 20 months. Five patients were responders in all seizure types, particularly GTCS and absences. Some patients had significant reductions in atonic seizures. Seizure reduction and severity improvement varied. Subgroup analysis for non-seizure outcome and complication were not provided.
Boon	Single center in Belgium	Prospective	3	All 3 responders had no side effects. All 3 had additional benefit from magnet stimulation (used by caregivers).
Buoni	Single center in Italy	Prospective Cohort	7	Age range was 6–28 years (median 17 years). A total of 38.4% had a ≥50% reduction in seizures. Some patients reported improved alertness, hyperactivity, appetite, and social communication. None had major complication; one had intractable headaches with increasing stimulation requiring decreased stim.
Casazza	Single center in Italy	Prospective cohort	4	None of them had >50% seizure reduction; fecal and urinary incontinence, associated with diarrhea, when the output current was increased. These side effects disappeared when there was a reduction in the intensity of the output current.
Cersósimo	Single center in Italy	Retrospective Cohort	46	Mean age at implant was 13 years, with a mean follow-up of 30 months. A total of 65% showed a ≥50% reduction in seizures. There were no significant differences between symptomatic and cryptogenic LGS patients or those with prior West syndrome. Improvement in behavior, cognition, and quality of life noted.
Cukiert	Single center in Brazil	Prospective Cohort	20	Age range was 5–12 years (mean 8.4 years). Mean follow-up was 32 months. A total of 17 out of 20 had a ≥50% seizure reduction. Quality of life and health measures improved in up to 50% of children. Attention improved in most children.
Cukiert (2022)	Single center in Brazil	Retrospective Cohort	10	Follow-up time after VNS ranged from 18 to 132 months (mean 52 months). Four patients initially responded to VNS.
Dibue	USA	Registry Analysis	564	Responder rates at 24 months were 57.5% in the LGS group and 61.5% in the non-LGS group. Median seizure frequency reduction at 24 months was 64.3% vs. 66.7% in the LGS vs. non-LGS group, respectively. VNS was most effective at reducing focal aware seizures, “other” seizures, generalized-onset non-motor seizures, and drop attacks. Significant regression from bilateral tonic–clonic response in LGS group. Within the 24 months of follow-up, there were 4 deaths in the LGS group and 5 deaths in the non-LGS group.
Elliott	Single center in the USA	Retrospective (prospective data collection)	24	Mean seizure reduction was 57.6% (43.0–72.2%). Median seizure reduction was 52.1%. Not different from the majority of the study group who had unidentified TRE causes (mean reduction: 57.0%). Non-seizure outcomes, etc., were not given for specific LGS groups.
Frost	6 centers in the USA	Retrospective Cohort	50 (46 had evaluable data at 1 month; 43 after 3 months, and 24 after 6 months)	Median age at implant was 13 years. At 1 month, median seizure reduction was 42%. At 3 and 6 months, reductions were 58.2% and 57.9%, respectively. Drop-attack seizures significantly decreased. QOL assessment (simple, invalidated, 5-point rating scale, which featured ratings of much worse, worse, same, better, and much better) was performed at 3 and 6 months. Improvement in alertness and verbal abilities in more patients than memory and mood. The least effect was shown on ambulation. The most common adverse events reported were voice alteration and coughing during stimulation. Other uncommon adverse events included increased drooling and behavioral changes. In total, 4% of patients had superficial wound infection; 10% had transient pain at the incision site; 44% had voice alteration/hoarseness. Moreover, 30% reported coughing, neck tingling (8%), nonspecific pain sensation (8%), shortness of breath during exertion (4%), decreased appetite (4%), hiccups (4%), and dyspepsia (4%). One (2%) patient reported dysphagia and insomnia, and another (2%) reported vomiting after stimulation started. One patient reported both ear pain and jaw pain as stimulation. Increased salivation (8%), worsening behavior or hyperactivity (6%).
Gurubani	Single center in the USA	Retrospective (prospective data collection)	13	Responder, 38.5%, partial responder(e (≥50% seizure reduction in 2 or less study periods) 23%, non-responder 38.5% in 24 months.
Hajnsek	Single center in Croatia	Retrospective	3	Mean age of 26.67 ± 5.77 13.34 ± 2.88; *p* = 0.02 (follow-up duration unknown); improvement of mood and the general quality of life was reported in all patients.
Hallbook	Single center in Sweden	Case Series	4	A total of 1 patient had a >25% seizure reduction. No changes in cognitive scales (BSID) in all 4 patients.
Hödl	Single center in Belgium	Prospective cohort	7	A total of 2/7 patients had >50% seizure reduction; no difference in pre-ictal HRV parameters between VNS responders and VNS non-responders could be found, but high frequency (HF) power, reflecting the parasympathetic tone increased significantly in the pre-ictal epoch in both VNS responders and VNS non-responders (*p* = 0.017, *p* = 0.004).
Hornig	Single center in the USA	Retrospective Cohort	6	A total of 5 out of 6 had a >90% seizure reduction. Assessed with a global evaluation score (10 cm horizontal line: central point no change; extreme left, considerable worse; extreme right, considerable improvement. Most (no specific n was given) had improvements in alertness, independence, and learning.
Hosain	Single center in the USA	Prospective Cohort	13	Age range 4–44 years. An amount of 52% seizure reduction at 6 months. Six patients had a >50% seizure reduction. No cognitive, behavioral, motor, or coordination worsening. A total of 3/13 had hoarseness; 3 had excessive coughing, 1 surgical debridement and antibiotics for incisional infection.
Kang	Single center in Korea	Retrospective cohort	7	A total of 6/7 were responders; the seizure frequency at the last follow-up (mean follow-up 38.8 months; range-29.7–49.7) showed a decrease of 57.2% (0% to 100%) on average. One patient achieved seizure free status. One patient with seizure worsening. There was no mortality or complications related to the VNS therapy except one case requiring ICU admission due to pneumonia. Comparing the results before and after VNS surgery, the VNS therapy also had a tendency to have a positive effect on quality of life (*p* = 0.066). Severity of illness: 4 improved, 3 unchanged; CGI-I: 2 marked improvement, 2 moderate, 4 minimal, and 1 unchanged.
Karceski	VNS patient registry	Retrospective	167	A total of 107 had >50% seizure reduction.
Katagiri	Single center in Japan	Retrospective	10	Age 10 years 8 months (range, 3–30 years); 6 had >50% seizure reduction in all residual seizure types after CC over 1 year follow-up; 2 were seizure free. Tonic seizures were the most common seizure type that was residual after CC. Responses were observed in five of the ten (50%) patients after VNS. Atypical absence seizures were less likely to be controlled with VNS (25% response rate). Myoclonic seizure was observed in four patients, two of whom were responders. None of the patients with residual drop attacks caused by atonic seizures responded to VNS. Patients who responded to VNS after CC could have conversations with others, while non-responders could not. After VNS, transient hoarseness was observed in two patients, and coughing only with magnet activation was observed in one patient.
Kim	Single center in Korea	Retrospective	14	The mean age 18.1 ± 4.9 years, and mean follow-up duration was 5.4 ± 2.2 years; one year after the procedure, 2 of the 14 patients showed a reduction in seizure frequency of more than 90%.
Kostov 2009	Single center in Norway	Retrospective Cohort	30	Median observation time was 52 months. Median seizure reduction was 60.6%. A total of 20/30 were responders. Best effects on atonic and tonic seizures. Additional improvements in postictal phase (16/30) and alertness (76.7%). Decrease in seizure duration from magnet use in 73.3% of the patients. Adverse effects 20/30, drooling and voice alteration 6/30. V cord paralysis in 1 patient (reverse following stimulator explanted). One with enuresis, etc. Most side effects transient and stimulation dependent.
Kostov 2023	Norwegian VNS quality registry	Retrospective Cohort	62	Median follow-up was 88 months. Seizure reduction at 6, 12, 24, 36, and 60 months was 18.8%, 31.3%, and 30.6%. Median seizure reduction for LGS was 40.6%. Highest reduction for atonic seizures (64.6%). Improved alertness and seizure severity noted. More DEE patients (total 105 patients; 62 of them had LGS) were reported to have greater improvement in ictal or postictal severity (43.8% vs. 28.3%, *p* = 0.006) and alertness (62.9% vs. 31.6%, *p* < 0.001) than patients without ID.
Labar	Single center in the USA	Prospective	5	Median seizure reduction 41% comparison of the rates of all seizures for the baseline month, with the rates of all seizures for the first 9 months of VNS treatment (2 out of 5 were responders) Variable reduction in different seizure types. Adverse events reported in one patient each were incisional infection, choking sensation and voice change, and coughing (noted by two patients). One patient discontinued VNS due to coughing.
Lundgren	Single center in Sweeden	Case Series	4	Two patients had a >50% seizure reduction. Improvement noted in seizure severity score and quality of life (linear visual analog scale: −100 to +100). Two patients had up to +50 at 16–18 months, one patient had 0, and one patient had QOL assessment up to 10–12 months, which was +10; premature current failure 2/4, aspiration ¼, tiredness ¼, increased salivation 2/4.
Mikati	Single center in Lebanon	Prospective cohort	6	Mean age 23 years; over mean follow-up of 1 year 11 months, 3 had >50% seizure reduction (−175% to 85% reduction). Seizure severity: 3/6 milder, 2/6 same, 1/6 stronger; 2/6 seizure duration decreased. For post-vagal nerve stimulation, the total group scored significantly higher in the social domain (*p* = 0.039) but LGS specific information was not available.
Nagarajan	Single center in Australia	Retrospective Cohort	6	A total of 5 out of 6 had a >50% seizure reduction. Reduction in seizure duration and severity, but the magnet was not useful. Total group outcome (*n* = 16) was given. On a three point scale, 12 of the 16 parents (entire group rather than LGS specific subgroup) reported that quality of life was definitely better and this correlated with improved seizure control.
Nakken	Single center in Norway	Retrospective	3	A total of 1/3 were responders. No other LGS-specific outcomes were available.
Orosz	Multiple centers in the European Union	Retrospective Cohort	123 (6 months), 146 (12 months), 87 (24 months)	At 6, 12, and 24 months, ≥50% seizure reduction in 28.5%, 32.9%, and 39.1% of patients, respectively. Higher responder rate with stable AEDs. Significant correlation between VNS total charge and response rate.
Parker	Single center in the UK	Retrospective Cohort	9	A total of 16 patients with epileptic encephalopathies. Median reduction for 9 children with LGS was 34% between 6 and 12 months. No significant improvement in EEG before and after implant. No significant difference in communication, living, or socialization domains for Vineland adaptive behavior scale. Significant improvement in perceived treatment side effects and general behavior.
Qiabi	Single center in Canada	Retrospective	5	A total of 3/5 were responders at 24 months. Non-seizure outcomes and adverse effects were not given.
Rossignol	Single center in Canada	Prospective	5	A total of 3/5 responders, 1 seizure free, maximal seizure effect 3/5 myoclonic and 1 with tonic.
Rychlicki	Italy	Prospective Cohort	9	A total of 34 patients. Significant better results in partial epilepsy, with and without drop attacks, than in LGS. Mean reduction in seizure frequency in LGS: 8% at 3 months, 33% at 12 months, and 50% at 2 years (not significant).
Shahwan	Single Center in Australia	Retrospective Cohort	9	Seven (77.7%) had a ≥50% seizure reduction. Significant reduction in daytime drop attacks and tonic seizures. Decrease in hospitalization for SE in some patients. Improved alertness in all responders.
Suller Marti	Single center in Canada	Retrospective Cohort	29	A total of 46 patients. Of the LGS group, 41.7% had an overall seizure reduction of 50% or more. Seizure reduction rate was 59% in LGS group and 64.7% in GGE group. A total of 96.6% of LGS patients required seizure-related hospital admissions before VNS implantation, 51.7% after implantation. Number of patients with >75% seizure reduction significantly higher in GGE group. GTC seizures reduced post-VNS, but no improvement in atypical absence seizures. Frequency of side effects similar in LGS vs. Non-LGS groups (62.1% vs 64.7%).
You	Multiple centers in South Korea	Retrospective Cohort	10	A total of 70% had a >50% seizure reduction; 20% had a >75% reduction. One patient experienced dyspnea while sleeping and one patient suffered from drooling.
Zamponi	Single center in Italy	Retrospective Cohort	14	A total of 3 out of 14 had a >50% seizure reduction. Improvement in seizure frequency and quality of life in some patients. Compared to baseline, after one year and three years of stimulation, no significant changes in cognitive level and adaptive behavior scores were observed in group level but 4 had clinically relevant improvement in adaptive behavior (at least 5 point increase in standard scores) mainly due to increased alertness and a better social reciprocity. Six patients had better QoL; this was independent of seizure outcome.

## Data Availability

Not applicable.

## Supplementary Materials

The following supporting information can be downloaded at https://www.mdpi.com/article/10.3390/children11080905/s1.
